# Supplementary figures and images for: LAP1 supports nuclear adaptability during constrained melanoma cell migration and invasion
Source: Nat Cell Biol. Author manuscript; Available in PMC 2023 Jan 24. (PMC9859759; doi:10.1038/s41556-022-01042-3)

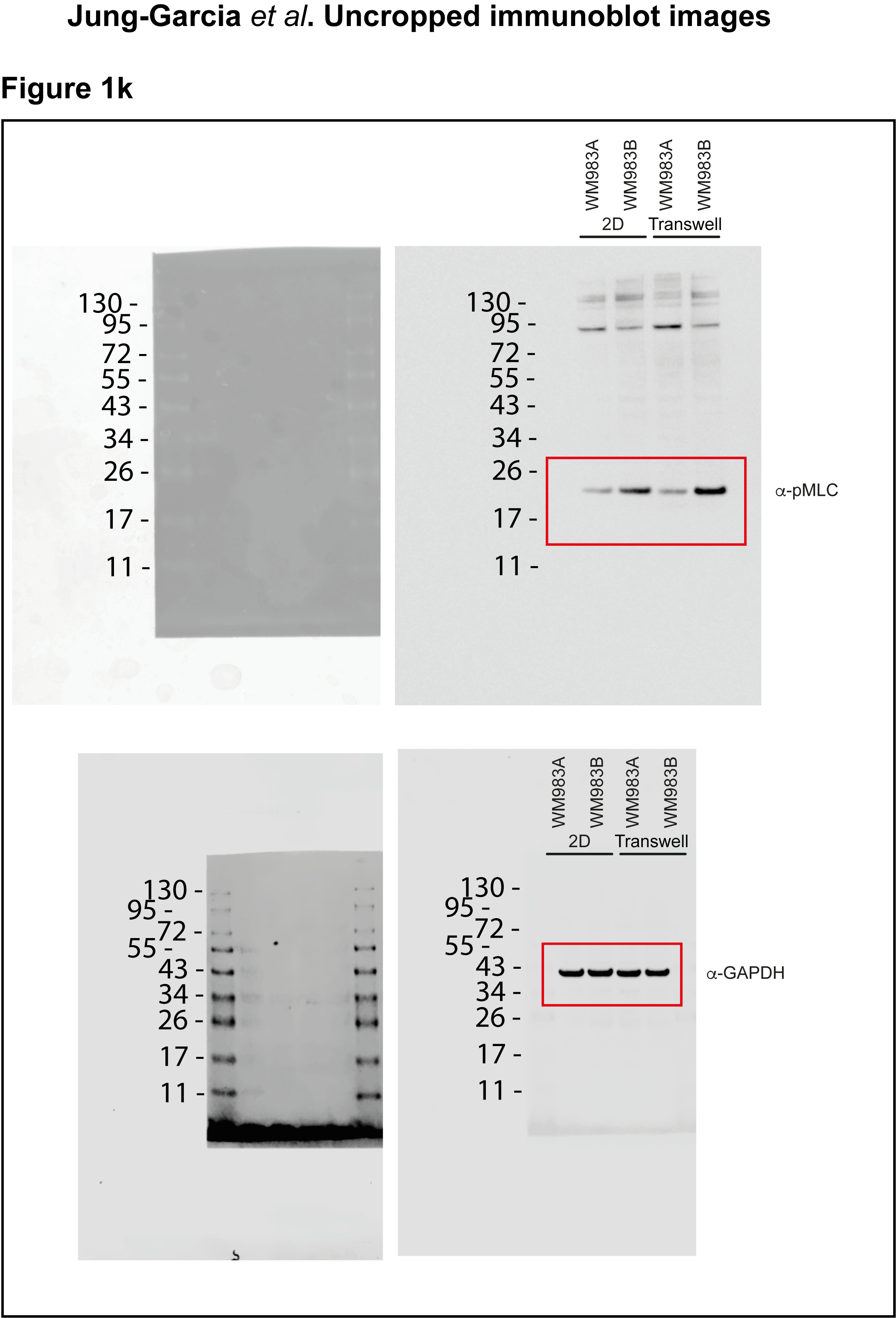

Supplement: Source data blot for Figure 1 [file EMS156666-supplement-Source_data_blot_for_Figure_1.tif]

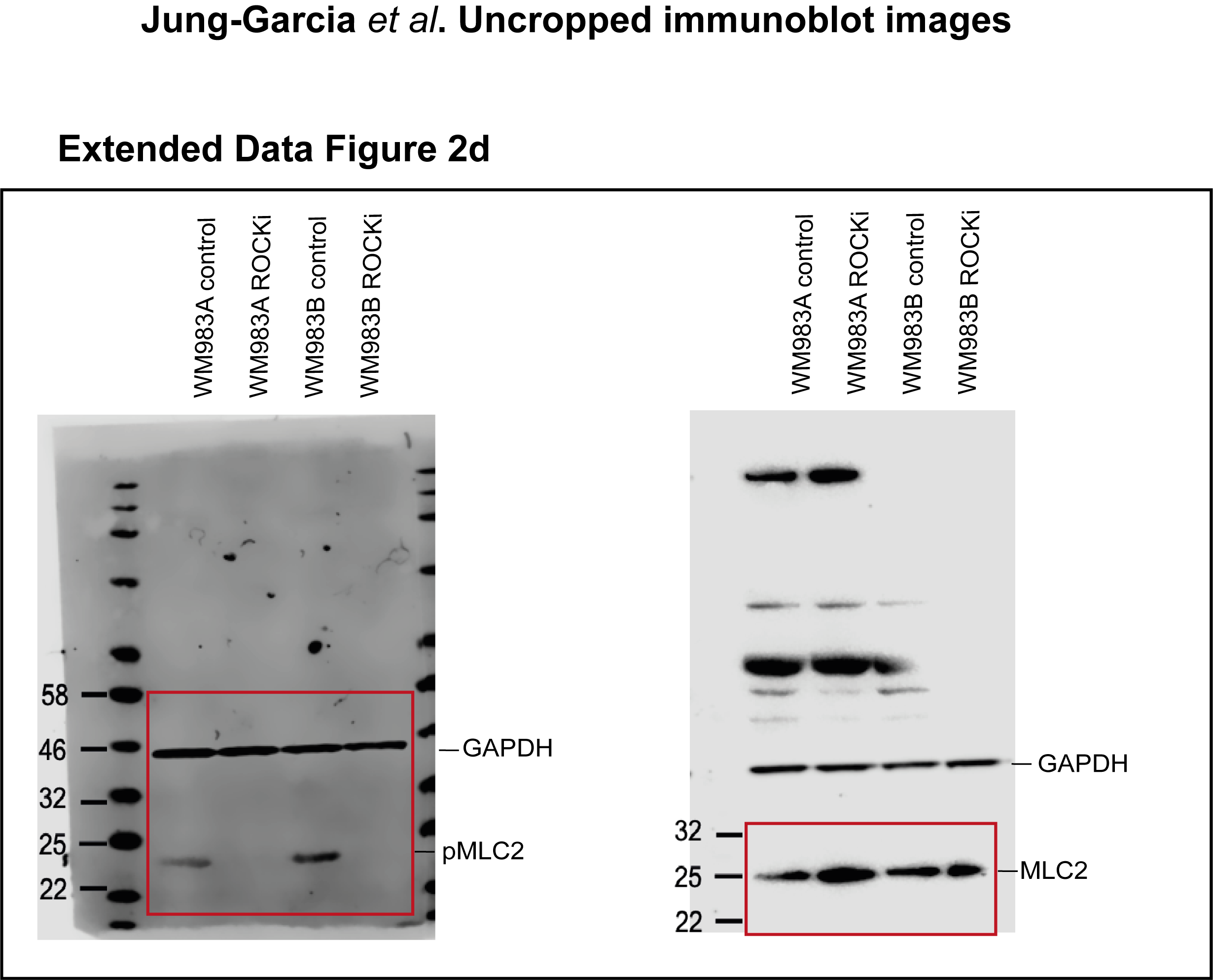

Supplement: Source data blot for Figure 2 [file EMS156666-supplement-Source_data_blot_for_Figure_2.tif]

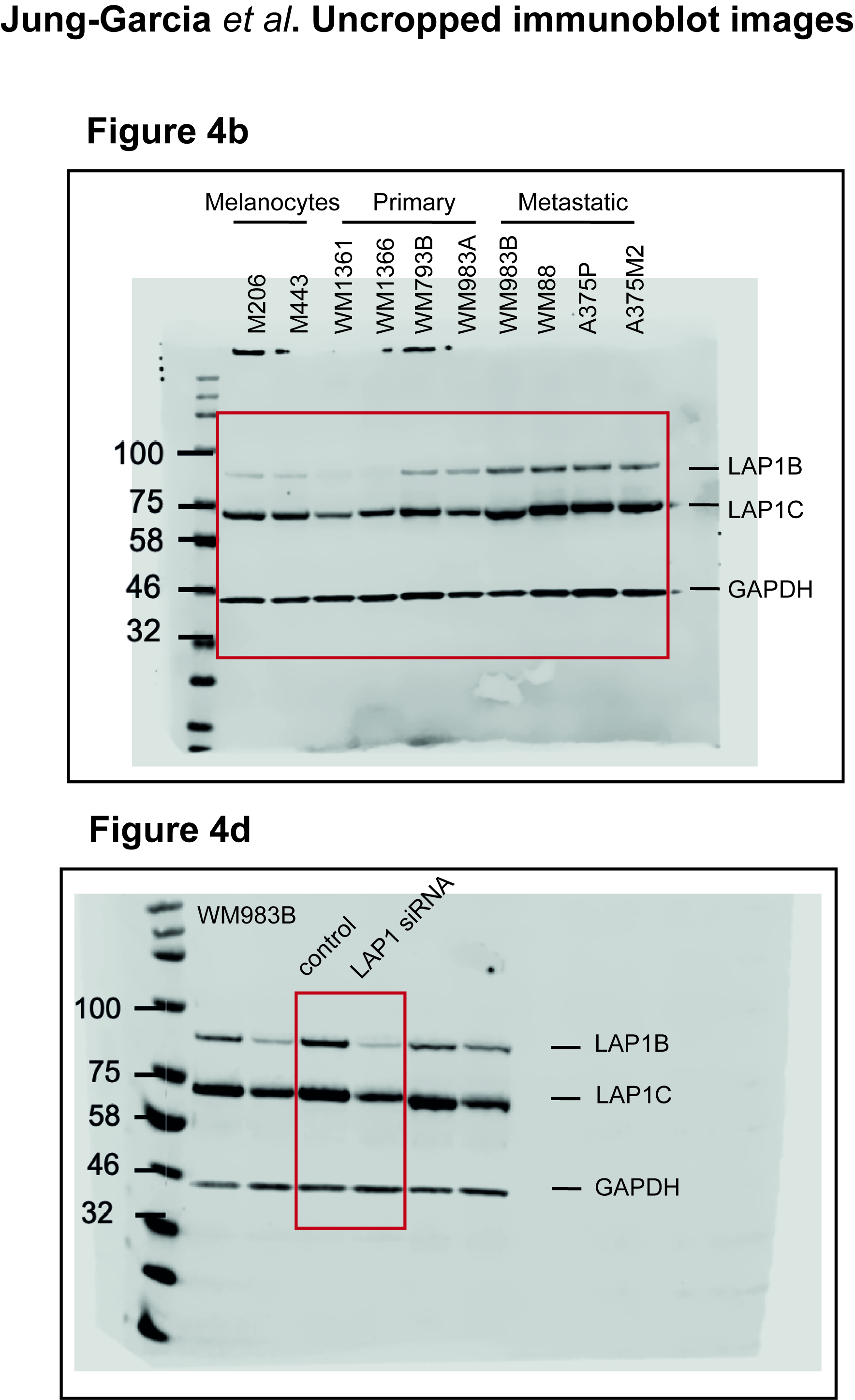

Supplement: Source data blot for Figure 4 [file EMS156666-supplement-Source_data_blot_for_Figure_4.tif]

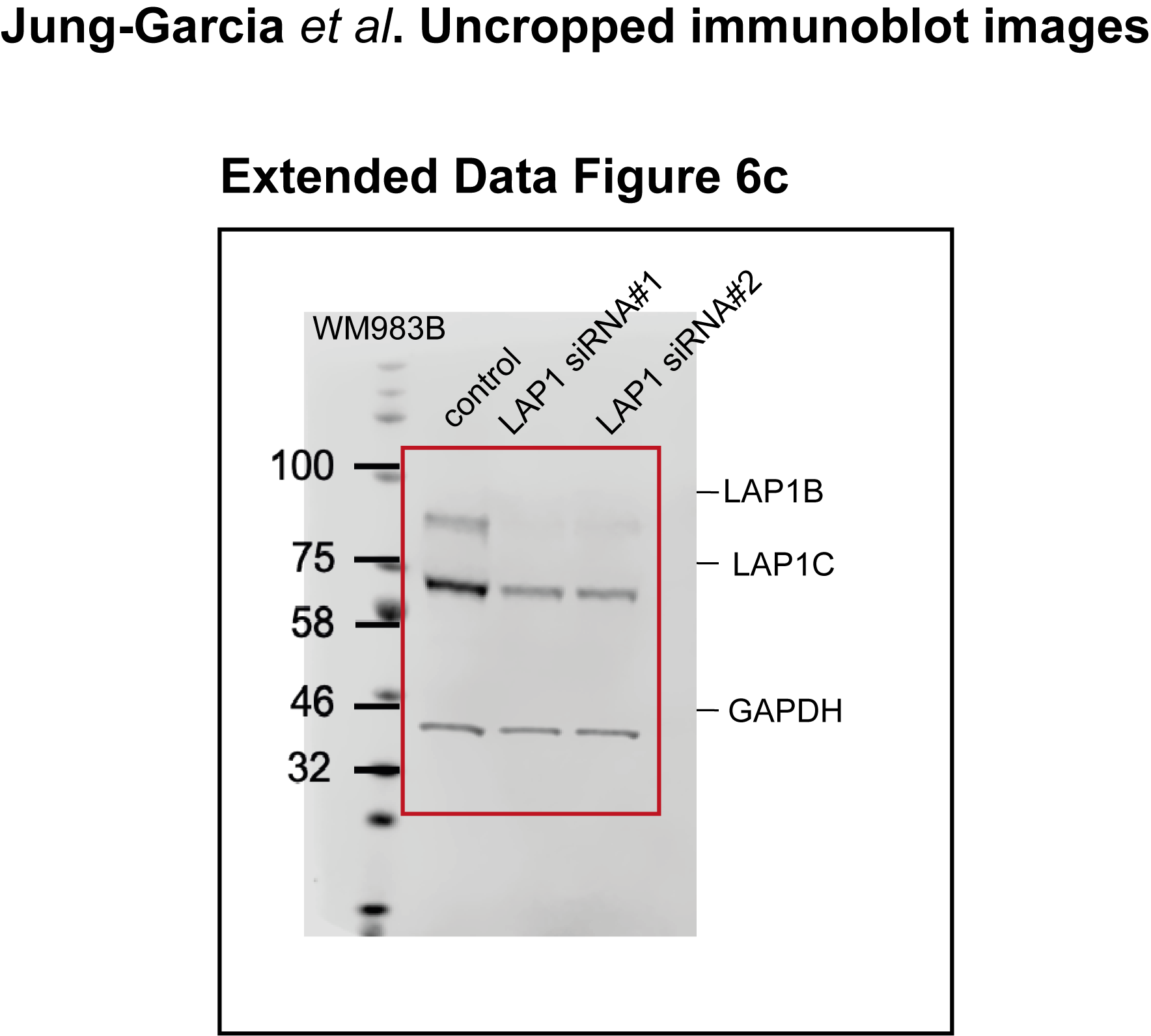

Supplement: Source data blot for Figure 6 [file EMS156666-supplement-Source_data_blot_for_Figure_6.tif]

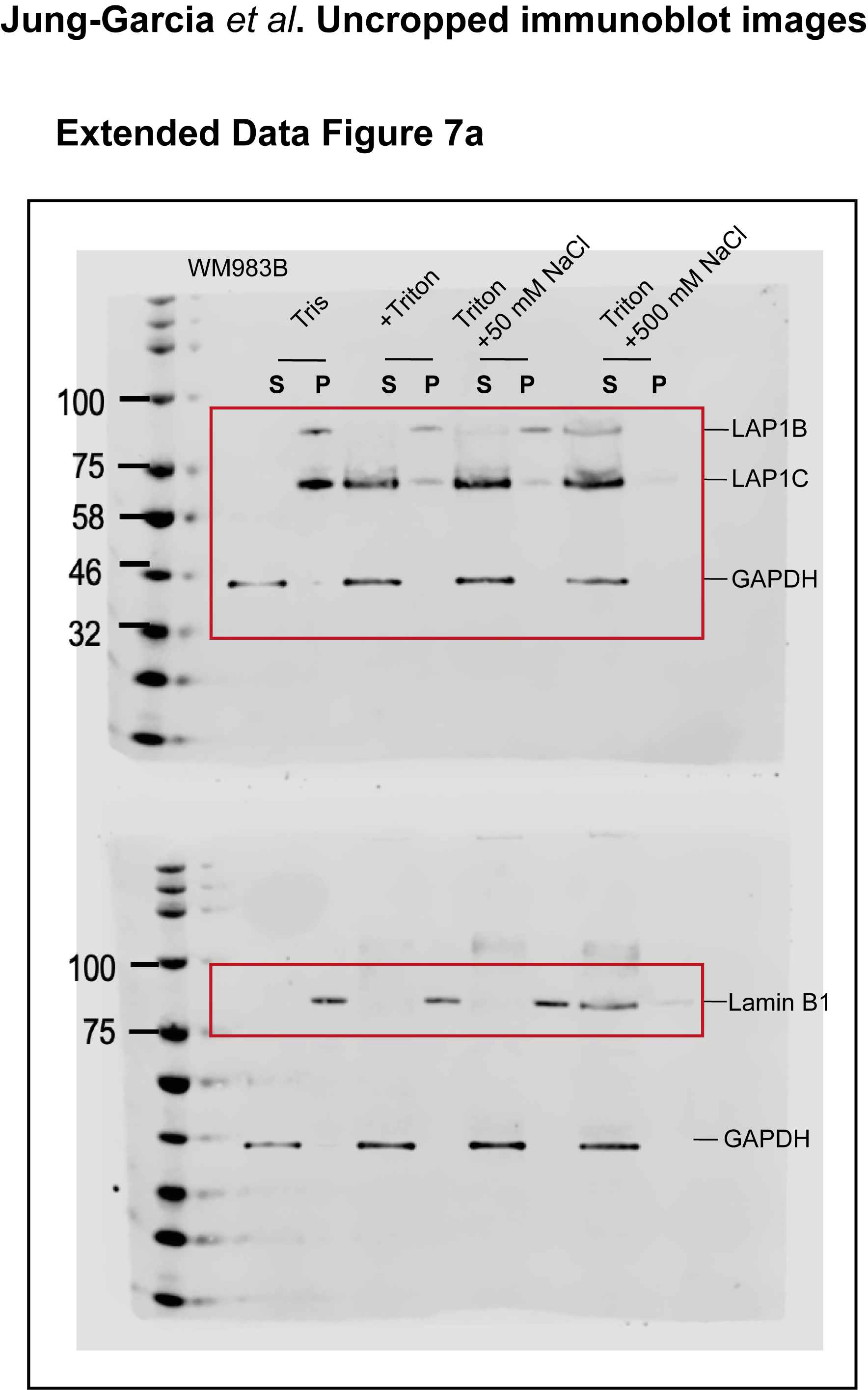

Supplement: Source data blot for Figure 7 [file EMS156666-supplement-Source_data_blot_for_Figure_7.tif]

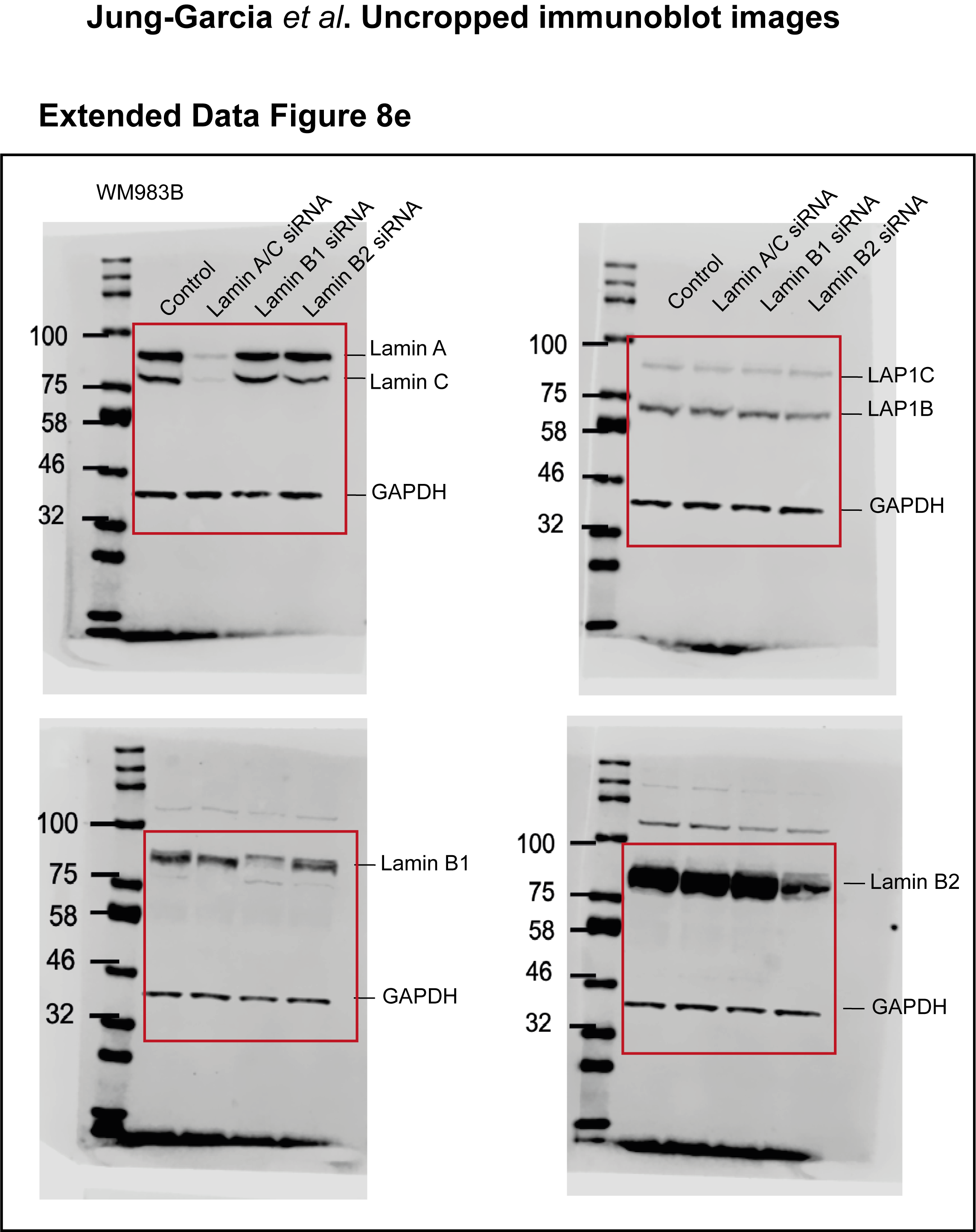

Supplement: Source data blot for Figure 8 [file EMS156666-supplement-Source_data_blot_for_Figure_8.tif]

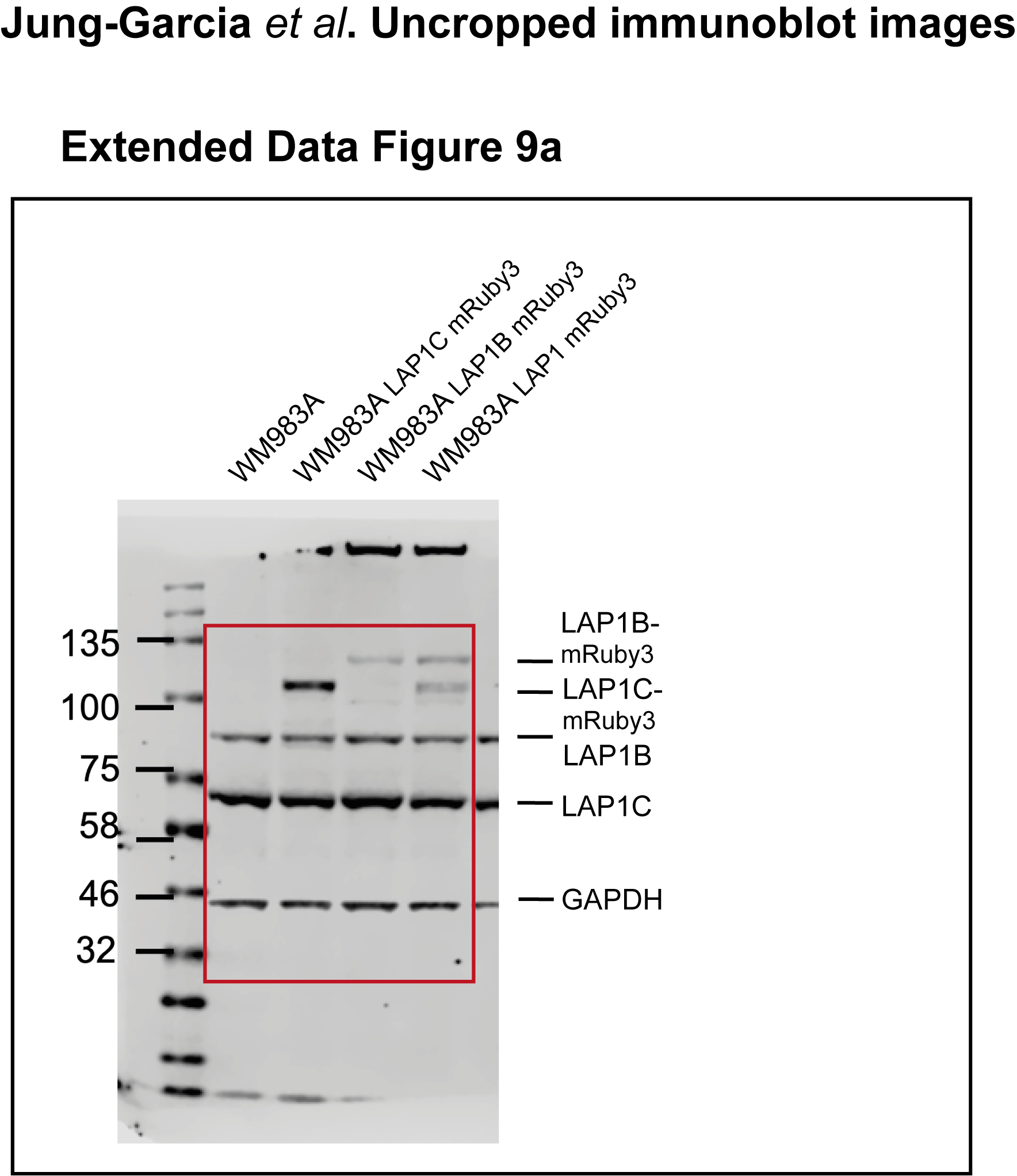

Supplement: Source data blot for Figure 9 [file EMS156666-supplement-Source_data_blot_for_Figure_9.tif]
