# Supplementary material for: LAP1 supports nuclear adaptability during constrained melanoma cell migration and invasion
Source: Nat Cell Biol. Author manuscript; Available in PMC 2023 Jan 24. (PMC9859759; doi:10.1038/s41556-022-01042-3)
Supplement: Supplementary Information [file EMS156666-supplement-Supplementary_Information.pdf]

Supplementary Material

LAP1 supports nuclear adaptability during constrained melanoma cell migration and invasion

Table of Contents

Supplementary Methods

Supplemental in vivo methods……………………………………………………………2

Supplemental electron microscopy processing steps…………………………………2

Supplemental immunohistochemistry processing steps………………………………3

SUPPLEMENTAL METHODS

In-vivo experiments.

Sample Size. No statistical methods were used to calculate sample size in mouse subcutaneous experiments. Sample was chosen based on standards in the field and previous experiments conducted in our laboratory[5,6,7,8,10,11]. The sample size was determined to be sufficient based on the size and consistency of the measurable differences between the groups. Test for normal distribution were not performed, the data was assumed to display a Gaussian distribution.

Randomisation. For in-vivo experiments, mice were randomly assigned to cages on arrival for injection with the given cancer cell types for both subcutaneous and intradermal mouse experiments. Beyond this, no randomisation was performed. Animals were identified by ear notching and animal cage. None of the studies required any treatment since tumour inoculation. Tumours were calipered and animals were sacrificed at the same time at the end point of the experiment. Note: For A375P intradermal injections two time points were considered (24 and 36 days) since we wanted to address changes in local invasion at early and late timepoints.

Blinding. During the in-vivo studies melanoma cell lines were injected subcutaneous/intradermally. Researchers were aware of the cell type implanted. Animals were kept under the same animal housing conditions (light, temperature, humidity and diet) and were regularly monitored body weight and tumour volume. Histological tissue sections and the corresponding IHC quantifications were performed using QuPath image analysis under the same conditions across the groups. Note: see IHC sections for parameters used in the analysis.

Power calculation for mouse experiment using NXG strain and A375P with LAP1 mutants was performed. We considered an effect size of (m1-m2): 0.5, variability of 0.35, significance level at least of 0.05, power score of 0.8 and two-sided test. We obtained n=9 per group. However, we increased to n=10 per group because intradermal injection risks ulceration which compromises the end point of the sample. At the end of the experiment, we did not observe any presence of ulceration, although they started to show red/glossy skin.

Electron Microscopy processing steps.

The samples were then processed using a Pelco BioWave Pro+ microwave (Ted Pella Inc, Redding, USA) and following a protocol adapted from the National Centre for Microscopy and Imaging Research protocol[57]. See Supplementary Table 17 for full BioWave program details. Each step was performed in the Biowave, except for the PB and water wash steps, which consisted of two washes on the bench followed by two washes in the Biowave without vacuum (at 250 W for 40 seconds). All the chemical incubations were performed in the Biowave for 14 minutes under vacuum in 2-minutes cycles alternating with/without 100W power. The SteadyTemp plate was set to 21ºC unless otherwise stated. In brief, the samples were fixed again in 2.5% (v/v) gluteraldehyde (TAAB) / 4% (v/v) formaldehyde in 0.1M PB. The cells were then stained with 2% (v/v) osmium tetroxide (TAAB) / 1.5% (v/v) potassium ferricyanide (Sigma), incubated in 1% (w/v) thiocarbohydrazide (Sigma) with SteadyTemp plate set to 40ºC, and further stained with 2% osmium tetroxide in ddH2O (w/v). The cells were then incubated in 1% aqueous uranyl acetate (Agar Scientific, Stansted, UK) with SteadyTemp plate set to 40ºC, and then washed in dH_2O with SteadyTemp set

to 40ºC. Samples were then stained with Walton's lead aspartate with SteadyTemp set to 50ºC, and dehydrated in a graded ethanol series (70%, 90%, and 100%, twice each), at 250 W for 40 seconds without vacuum. Exchange into Durcupan ACM® resin (Sigma) was performed in 50% resin in ethanol, followed by 4 pure Durcupan steps, at 250 W for 3 minutes, with vacuum cycling (on/off at 30-seconds intervals), before embedding at 60ºC for 48 hours. Blocks were trimmed to a small trapezoid, excised from the resin block, and attached to a serial block-face scanning electron microscopy (SBF SEM) specimen holder using conductive epoxy resin. Prior to commencement of a SBF SEM imaging run, the sample were coated with a 2 nm layer of platinum to further enhance conductivity. SBF-SEM data was collected using a 3View2XP (Gatan, Pleasanton, CA) attached to a Sigma VP SEM (Carl Zeiss Ltd, Cambridge, UK). Inverted backscattered electron images were acquired through the entire extent of the region of interest. For each of the 50-nm slices needed to image the cells in their whole volume, a low-resolution overview image (horizontal frame width 103 µm; pixel size of 40 nm; using a 2 µseconds dwell time) and a high-resolution image of the cell of interest (horizontal frame width 32 and 39 µm respectively; pixel size of 8 nm; using a 2 µseconds dwell time) were acquired. The SEM was operated in high vacuum with focal charge compensation on (70%). The 30 µm aperture was used, at an accelerating voltage of 1.8 kV. Only minor adjustments in image alignment were needed and were done using the TrakEM2 plug-in of the FIJI framework[58].

Immunohistochemistry processing steps.
Samples were sectioned (3-4 µm thick) and dried for one hour at 65°C. Next, samples were deparaffined and rehydrated and endogenous peroxidase activity was blocked with 3% H_2O_2 in ethanol absolute for 10 minutes. Heat-induced epitope retrieval was carried out using 1:100 pH 6 Citrate Buffer H-3300 for 10 minutes at 100°C in a Biocare Decloaking Chamber (DC2012). Incubation with primary antibody in Zytomed antibody diluent was carried out for 40 minutes. Incubation with secondary antibody polymer conjugated (ImmPRESS Polymer Reagent) was carried out for 45 minutes. Incubation with Vector VIP HRP substrate chromogen was done for up to 10 minutes. All reagents used for detection were from VECTASTAIN ABC-HRP Kit (PK-4000). All samples were counterstained with haematoxylin. Lastly, samples were dehydrated, and slides were mounted. Reagents were used at RT in humidified slide chambers. Primary antibodies were: LAP1 (1:100; #21459-1-AP) from Proteintech, CITED1 (1:200; #ab87978) from Abcam, GFP (1:1500, #A-11122) from ThermoFisher, SOX10 [EPR4007] (1:500; #ab155279) from Abcam. Whole section images were obtained from each sample using a NanoZoomer S210 slide scanner (Hamamatsu, Japan). Image analysis was done using QuPath software[59]. For LAP1 expression, Positive cell detection was carried out and threshold to the intensity scores (0,1, 2, 3) was applied. Then, QuPath software was trained to differentiate tumour cells from stroma, staining was graded semiquantitatively and H-scores were calculated as previously described[9]. Invading cells were scored at the distal invasive front (DIF) creating manual annotations and performing Cell Detection using Qupath. Background radius was reduced to 2 µm, Minimum area increased to 50 µm and Intensity threshold increased at 0.15. For IHC images, DIF regions were also created manually, and Positive Cell Detection was applied with a single threshold. The number of positive cells per area (mm^2) was calculated as represented in graphs.
