# Supplementary material for: LAP1 supports nuclear adaptability during constrained melanoma cell migration and invasion
Source: Nat Cell Biol. Author manuscript; Available in PMC 2023 Jan 24. (PMC9859759; doi:10.1038/s41556-022-01042-3)
Supplement: Supplementary Legends and Information [file EMS156666-supplement-Supplementary_Legends_and_Information.docx]

**Supplementary Tables**

**Supplementary Table 1. Signalling regulation gene sets enriched in A375M2.** The table shows the gene set name, gene set size, enrichment score (ES), normalised enrichment score (NES), nominal p-value for the enrichment score calculated by phenotype-based permutation test, false discovery rate (FDR q-value), familywise-error rate (FWER p-value), and the position in the ranked list at which the maximum enrichment score occurred (RANK MAX) of signalling regulation gene sets enriched in highly metastatic melanoma A375M2 cells.

**Supplementary Table 2. Nuclear membrane gene sets enriched in A375M2.** The table shows the gene set name, gene set size, enrichment score (ES), normalised enrichment score (NES), nominal p-value for the enrichment score calculated by phenotype-based permutation test, false discovery rate (FDR q-value), familywise-error rate (FWER p-value), and the position in the ranked list at which the maximum enrichment score occurred (RANK MAX) of nuclear membrane gene sets enriched in highly metastatic melanoma A375M2 cells.

**Supplementary Table 3. Cell division gene sets enriched in A375M2.** The table shows the gene set name, gene set size, enrichment score (ES), normalised enrichment score (NES), nominal p-value for the enrichment score calculated by phenotype-based permutation test, false discovery rate (FDR q-value), familywise-error rate (FWER p-value), and the position in the ranked list at which the maximum enrichment score occurred (RANK MAX) of cell division gene sets enriched in highly metastatic melanoma A375M2 cells.

**Supplementary Table 4. Organelle organisation gene sets enriched in A375M2.** The table shows the gene set name, gene set size, enrichment score (ES), normalised enrichment score (NES), nominal p-value for the enrichment score calculated by phenotype-based permutation test, false discovery rate (FDR q-value), familywise-error rate (FWER p-value), and the position in the ranked list at which the maximum enrichment score occurred (RANK MAX) of organelle organisation gene sets enriched in highly metastatic melanoma A375M2 cells.

**Supplementary Extended Data Table 5. Cellular localisation gene sets enriched in A375M2.** The table shows the gene set name, gene set size, enrichment score (ES), normalised enrichment score (NES), nominal p-value for the enrichment score calculated by phenotype-based permutation test, false discovery rate (FDR q-value), familywise-error rate (FWER p-value), and the position in the ranked list at which the maximum enrichment score occurred (RANK MAX) of cellular localisation gene sets enriched in highly metastatic melanoma A375M2 cells.

**Supplementary Table 6. Cellular development gene sets enriched in A375M2.** The table shows the gene set name, gene set size, enrichment score (ES), normalised enrichment score (NES), nominal p-value for the enrichment score calculated by phenotype-based permutation test, false discovery rate (FDR q-value), familywise-error rate (FWER p-value), and the position in the ranked list at which the maximum enrichment score occurred (RANK MAX) of cellular development gene sets enriched in highly metastatic melanoma A375M2 cells.

**Supplementary Table 7. Cytoskeleton organisation gene sets enriched in A375M2.** The table shows the gene set name, gene set size, enrichment score (ES), normalised enrichment score (NES), nominal p-value for the enrichment score calculated by phenotype-based permutation test, false discovery rate (FDR q-value), familywise-error rate (FWER p-value), and the position in the ranked list at which the maximum enrichment score occurred (RANK MAX) of cytoskeleton organisation gene sets enriched in highly metastatic melanoma A375M2 cells.

**Supplementary Table 8. Cellular movement gene sets enriched in A375M2.** The table shows the gene set name, gene set size, enrichment score (ES), normalised enrichment score (NES), nominal p-value for the enrichment score calculated by phenotype-based permutation test, false discovery rate (FDR q-value), familywise-error rate (FWER p-value), and the position in the ranked list at which the maximum enrichment score occurred (RANK MAX) of cellular movement gene sets enriched in highly metastatic melanoma A375M2 cells.

**Supplementary Table 9. Cellular transport gene sets enriched in A375M2.** The table shows the gene set name, gene set size, enrichment score (ES), normalised enrichment score (NES), nominal p-value for the enrichment score calculated by phenotype-based permutation test, false discovery rate (FDR q-value), familywise-error rate (FWER p-value), and the position in the ranked list at which the maximum enrichment score occurred (RANK MAX) of cellular transport gene sets enriched in highly metastatic melanoma A375M2 cells.

**Supplementary Table 10. Membrane remodelling gene sets enriched in A375M2.**

The table shows the gene set name, gene set size, enrichment score (ES), normalised enrichment score (NES), nominal p-value for the enrichment score calculated by phenotype-based permutation test, false discovery rate (FDR q-value), familywise-error rate (FWER p-value), and the position in the ranked list at which the maximum enrichment score occurred (RANK MAX) of membrane remodelling gene sets enriched in highly metastatic melanoma A375M2 cells.

**Supplementary Table 11. Nuclear matrix gene sets enriched in A375M2.** The table shows the gene set name, gene set size, enrichment score (ES), normalised enrichment score (NES), nominal p-value for the enrichment score calculated by phenotype-based permutation test, false discovery rate (FDR q-value), familywise-error rate (FWER p-value), and the position in the ranked list at which the maximum enrichment score occurred (RANK MAX) of nuclear matrix gene sets enriched in highly metastatic melanoma A375M2 cells.

**Supplementary Table 12. Biosynthesis gene sets enriched in A375M2.** The table shows the gene set name, gene set size, enrichment score (ES), normalised enrichment score (NES), nominal p-value for the enrichment score calculated by phenotype-based permutation test, false discovery rate (FDR q-value), familywise-error rate (FWER p-value), and the position in the ranked list at which the maximum enrichment score occurred (RANK MAX) of biosynthesis gene sets enriched in highly metastatic melanoma A375M2 cells.

**Supplementary Table 13. Clinical information for primary melanoma patients.** The table shows the average patient age ± the standard deviation, the number and percentage of patients according to gender, and the number and percentage of tumours according to location for human melanoma patients in cohorts A and B.

**Supplementary Table 14. Clinical information for metastatic melanoma patients.** The table shows the average patient age ± the standard deviation, the number and percentage of patients according to gender, and the number and percentage of metastases according to location for human melanoma patients in cohorts A and B.

**Supplementary Table 15. List of PCR primer sequences.** The table shows the PCR primer name and nucleotide sequence.

**Supplementary Table 16. List of siRNA sequences. details.** The table shows the siRNA target and siRNA oligo sequence.

**Supplementary Table 17. List of BioWave program details.** The table shows a description of the BioWave program steps, step duration, power, and vacuum cycle details.

**Supplementary Video Legends**

**Supplementary Video 1. Intact nuclear envelope bleb by SBF SEM.** Representative SBF SEM reconstruction of the nucleus of a metastatic melanoma WM983B cell with an intact nuclear envelope bleb. Scale bar, 5 μm.

**Supplementary Video 2. Intact nuclear envelope bleb by CLEM.** Representative correlative live imaging prior to fixation for CLEM of the nucleus of a metastatic melanoma WM983B cell stably expressing GFP-NLS (green) with an intact nuclear envelope bleb. Time interval: 6 min. Scale bar, 5 μm.

**Supplementary Video 3. Ruptured nuclear envelope bleb by SBF SEM.** Representative SBF SEM reconstruction of the nucleus of a metastatic melanoma WM983B cell with a ruptured nuclear envelope bleb. Scale bar, 5 μm.

**Supplementary Video 4. Ruptured nuclear envelope bleb by CLEM.** Representative correlative live imaging prior to fixation for CLEM movie of the nucleus of a metastatic melanoma WM983B cell stably expressing GFP-NLS (green) with a ruptured nuclear envelope bleb. Time interval: 6 min. Scale bar, 5 μm.

**Supplementary Video 5. Nuclear envelope rupture in primary melanoma cell.** Representative movie of nuclear envelope rupture in a WM983A cell stably expressing GFP-NLS (green). Time interval: 5 hours. Scale bar, 10 μm.

**Supplementary Video 6. Nuclear envelope rupture in metastatic melanoma cell.** Representative movie of nuclear envelope rupture in a WM983B cell stably expressing GFP-NLS (green). Time interval: 5 hours. Scale bar, 10 μm.

**Supplementary Video 7. FRAP of LAP1 isoforms in metastatic melanoma cell.** Representative movie of FRAP in a WM983B nucleus stably co-expressing LAP1B-GFP (LAP1 M122A) (green) and LAP1C-mRuby3 (magenta) and with a nuclear envelope bleb. FRAP was measured at the main nuclear envelope and at the bleb. Time interval: 200 seconds. Scale bar, 5 μm. Merge.

**Supplementary Video 8. FRAP of LAP1B in metastatic melanoma cell.** Representative movie of FRAP in a WM983B nucleus stably co-expressing LAP1B-GFP (LAP1 M122A) (green) and LAP1C-mRuby3 (magenta) and with a nuclear envelope bleb. FRAP was measured at the main nuclear envelope and at the bleb. Time interval: 200 seconds. Scale bar, 5 μm. Green channel displayed.

**Supplementary Video 9. FRAP of LAP1C in metastatic melanoma cell.** Representative movie of FRAP in a WM983B nucleus stably co-expressing LAP1B-GFP (M122A) (green) and LAP1C-mRuby3 (magenta) and with a nuclear envelope bleb. FRAP was measured at the main nuclear envelope and at the bleb. Time interval: 200 seconds. Scale bar, 5 μm. Magenta channel displayed.

**Supplementary Material**

**LAP1 supports nuclear adaptability during constrained melanoma cell migration and invasion**

**Table of Contents**

Supplementary Methods

Supplemental in vivo methods………………………………………………………………2

Supplemental electron microscopy processing steps……………………………………2

Supplemental immunohistochemistry processing steps…………………………………3

**SUPPLEMENTAL METHODS**

**In-vivo experiments.**

Sample Size. No statistical methods were used to calculate sample size in mouse subcutaneous experiments. Sample was chosen based on standards in the field and previous experiments conducted in our laboratory^5,6,7,8,10,11^. The sample size was determined to be sufficient based on the size and consistency of the measurable differences between the groups. Test for normal distribution were not performed, the data was assumed to display a Gaussian distribution.

Randomisation. For in-vivo experiments, mice were randomly assigned to cages on arrival for injection with the given cancer cell types for both subcutaneous and intradermal mouse experiments. Beyond this, no randomisation was performed. Animals were identified by ear notching and animal cage. None of the studies required any treatment since tumour inoculation. Tumours were calipered and animals were sacrificed at the same time at the end point of the experiment. Note: For A375P intradermal injections two time points were considered (24 and 36 days) since we wanted to address changes in local invasion at early and late timepoints.

Blinding. During the in-vivo studies melanoma cell lines were injected subcutaneous/intradermally. Researchers were aware of the cell type implanted. Animals were kept under the same animal housing conditions (light, temperature, humidity and diet) and were regularly monitored body weight and tumour volume. Histological tissue sections and the corresponding IHC quantifications were performed using QuPath image analysis under the same conditions across the groups. Note: see IHC sections for parameters used in the analysis.

Power calculation for mouse experiment using NXG strain and A375P with LAP1 mutants was performed. We considered an effect size of (m1-m2): 0.5, variability of 0.35, significance level at least of 0.05, power score of 0.8 and two-sided test. We obtained n=9 per group. However, we increased to n=10 per group because intradermal injection risks ulceration which compromises the end point of the sample. At the end of the experiment, we did not observe any presence of ulceration, although they started to show red/glossy skin.

**Electron Microscopy processing steps.**

The samples were then processed using a Pelco BioWave Pro+ microwave (Ted Pella Inc, Redding, USA) and following a protocol adapted from the National Centre for Microscopy and Imaging Research protocol^57^. See Supplementary Table 17 for full BioWave program details. Each step was performed in the Biowave, except for the PB and water wash steps, which consisted of two washes on the bench followed by two washes in the Biowave without vacuum (at 250 W for 40 seconds). All the chemical incubations were performed in the Biowave for 14 minutes under vacuum in 2-minutes cycles alternating with/without 100W power. The SteadyTemp plate was set to 21ºC unless otherwise stated. In brief, the samples were fixed again in 2.5% (v/v) gluteraldehyde (TAAB) / 4% (v/v) formaldehyde in 0.1M PB. The cells were then stained with 2% (v/v) osmium tetroxide (TAAB) / 1.5% (v/v) potassium ferricyanide (Sigma), incubated in 1% (w/v) thiocarbohydrazide (Sigma) with SteadyTemp plate set to 40ºC, and further stained with 2% osmium tetroxide in ddH2O (w/v). The cells were then incubated in 1% aqueous uranyl acetate (Agar Scientific, Stansted, UK) with SteadyTemp plate set to 40ºC, and then washed in dH_2_O with SteadyTemp set to 40ºC. Samples were then stained with Walton's lead aspartate with SteadyTemp set to 50ºC, and dehydrated in a graded ethanol series (70%, 90%, and 100%, twice each), at 250 W for 40 seconds without vacuum. Exchange into Durcupan ACM® resin (Sigma) was performed in 50% resin in ethanol, followed by 4 pure Durcupan steps, at 250 W for 3 minutes, with vacuum cycling (on/off at 30-seconds intervals), before embedding at 60ºC for 48 hours. Blocks were trimmed to a small trapezoid, excised from the resin block, and attached to a serial block-face scanning electron microscopy (SBF SEM) specimen holder using conductive epoxy resin. Prior to commencement of a SBF SEM imaging run, the sample were coated with a 2 nm layer of platinum to further enhance conductivity. SBF-SEM data was collected using a 3View2XP (Gatan, Pleasanton, CA) attached to a Sigma VP SEM (Carl Zeiss Ltd, Cambridge, UK). Inverted backscattered electron images were acquired through the entire extent of the region of interest. For each of the 50-nm slices needed to image the cells in their whole volume, a low-resolution overview image (horizontal frame width 103 µm; pixel size of 40 nm; using a 2 µseconds dwell time) and a high-resolution image of the cell of interest (horizontal frame width 32 and 39 µm respectively; pixel size of 8 nm; using a 2 µseconds dwell time) were acquired. The SEM was operated in high vacuum with focal charge compensation on (70%). The 30 µm aperture was used, at an accelerating voltage of 1.8 kV. Only minor adjustments in image alignment were needed and were done using the TrakEM2 plug-in of the FIJI framework^58^.

**Immunohistochemistry processing steps.**

Samples were sectioned (3-4 μm thick) and dried for one hour at 65°C. Next, samples were deparaffined and rehydrated and endogenous peroxidase activity was blocked with 3% H_2_O_2_ in ethanol absolute for 10 minutes. Heat-induced epitope retrieval was carried out using 1:100 pH 6 Citrate Buffer H-3300 for 10 minutes at 100°C in a Biocare Decloaking Chamber (DC2012). Incubation with primary antibody in Zytomed antibody diluent was carried out for 40 minutes. Incubation with secondary antibody polymer conjugated (ImmPRESS Polymer Reagent) was carried out for 45 minutes. Incubation with Vector VIP HRP substrate chromogen was done for up to 10 minutes. All reagents used for detection were from VECTASTAIN ABC-HRP Kit (PK-4000). All samples were counterstained with haematoxylin. Lastly, samples were dehydrated, and slides were mounted. Reagents were used at RT in humidified slide chambers. Primary antibodies were: LAP1 (1:100; #21459-1-AP) from Proteintech, CITED1 (1:200; #ab87978) from Abcam, GFP (1:1500, #A-11122) from ThermoFisher, SOX10 [EPR4007] (1:500; #ab155279) from Abcam. Whole section images were obtained from each sample using a NanoZoomer S210 slide scanner (Hamamatsu, Japan). Image analysis was done using QuPath software^59^. For LAP1 expression, Positive cell detection was carried out and threshold to the intensity scores (0,1, 2, 3) was applied. Then, QuPath software was trained to differentiate tumour cells from stroma, staining was graded semiquantitatively and H-scores were calculated as previously described^9^. Invading cells were scored at the distal invasive front (DIF) creating manual annotations and performing Cell Detection using Qupath. Background radius was reduced to 2 μm, Minimum area increased to 50 μm and Intensity threshold increased at 0.15. For IHC images, DIF regions were also created manually, and Positive Cell Detection was applied with a single threshold. The number of positive cells per area (mm^2^) was calculated as represented in graphs.
