# Supplementary material for: LAP1 supports nuclear adaptability during constrained melanoma cell migration and invasion
Source: Nat Cell Biol. 2023 Jan 9;25(1):108–19. doi: 10.1038/s41556-022-01042-3 (PMC9859759; doi:10.1038/s41556-022-01042-3)
Supplement: Supplementary file 2 [file 41556_2022_1042_MOESM2_ESM.pdf]

nature portfolio

Corresponding author(s): J.G.Carlton & V.Sanz-Moreno

Last updated by author(s): Oct 1, 2022

Reporting Summary

Nature Portfolio wishes to improve the reproducibility of the work that we publish. This form provides structure for consistency and transparency in reporting. For further information on Nature Portfolio policies, see our Editorial Policies and the Editorial Policy Checklist.

Statistics

For all statistical analyses, confirm that the following items are present in the figure legend, table legend, main text, or Methods section.

n/a	Confirmed	
☐	☒	The exact sample size (*n*) for each experimental group/condition, given as a discrete number and unit of measurement
☐	☒	A statement on whether measurements were taken from distinct samples or whether the same sample was measured repeatedly
☐	☒	The statistical test(s) used AND whether they are one- or two-sided<br>*Only common tests should be described solely by name; describe more complex techniques in the Methods section.*
☒	☐	A description of all covariates tested
☐	☒	A description of any assumptions or corrections, such as tests of normality and adjustment for multiple comparisons
☐	☒	A full description of the statistical parameters including central tendency (e.g. means) or other basic estimates (e.g. regression coefficient) AND variation (e.g. standard deviation) or associated estimates of uncertainty (e.g. confidence intervals)
☐	☒	For null hypothesis testing, the test statistic (e.g. *F*, *t*, *r*) with confidence intervals, effect sizes, degrees of freedom and *P* value noted<br>*Give P values as exact values whenever suitable.*
☒	☐	For Bayesian analysis, information on the choice of priors and Markov chain Monte Carlo settings
☒	☐	For hierarchical and complex designs, identification of the appropriate level for tests and full reporting of outcomes
☒	☐	Estimates of effect sizes (e.g. Cohen's *d*, Pearson's *r*), indicating how they were calculated

Our web collection on statistics for biologists contains articles on many of the points above.

Software and code

Policy information about availability of computer code

Data collection	Excluding manufacturer's software required to run the instruments described in the methods section, no custom code was required to acquire any data in this manuscript.
Data analysis	The following software packages were used to analyse data in this manuscript. qPCR - ViiA 7 Real-Time PCR System; Western blotting - LiCOR Image Studio Version 5.2; Confocal fluorescence microscopy - ImageJ version 2.1.0/1.53c; Immunohistochemistry - QuPath version 0.2.1; Gene expression analysis - GSEA software (http://www.broadinstitute.org/gsea/index.jsp) and heatmaps using MeV (4.9.0, http://mev.tm4.org/); Statistics - GraphPad Prism (GraphPad Software, Inc) version 9.0.2 (134).

For manuscripts utilizing custom algorithms or software that are central to the research but not yet described in published literature, software must be made available to editors and reviewers. We strongly encourage code deposition in a community repository (e.g. GitHub). See the Nature Portfolio guidelines for submitting code & software for further information.

Data

Policy information about availability of data

All manuscripts must include a data availability statement. This statement should provide the following information, where applicable:

- Accession codes, unique identifiers, or web links for publicly available datasets
- A description of any restrictions on data availability
- For clinical datasets or third party data, please ensure that the statement adheres to our policy

Gene expression datasets re-analysed in this study are available from NCBI GEO under accession numbers: GSE23764, GSE4840, GSE4841, GSE4843, GSE4570 and GSE7553, GSE46517 & GSE8401. The datasets generated during and/or analysed during the current study are available from the corresponding author on reasonable request.

Field-specific reporting

Please select the one below that is the best fit for your research. If you are not sure, read the appropriate sections before making your selection.

☒ Life sciences ☐ Behavioural & social sciences ☐ Ecological, evolutionary & environmental sciences

For a reference copy of the document with all sections, see nature.com/documents/nr-reporting-summary-flat.pdf

Life sciences study design

All studies must disclose on these points even when the disclosure is negative.

Sample size	No statistical methods were used to calculate sample size. Sample size was chosen based on standards in the field and previous experiments conducted in our laboratory (Sanz-Moreno et al, Cell 2008; Sanz-Moreno et al, Cancer Cell 2011; Orgaz et al, Nat Commun 2014; Cantelli et al, Curr Biol 2015; Herraiz et al, J Natl Cancer Inst 2015; Georgouli et al, Cell 2019; Orgaz et al, Cancer Cell 2020). The sample size was determined to be sufficient based on the size and consistency of the measurable differences between the groups. Tests for normal distribution were not performed, the data was assumed to display a Gaussian distribution.
Data exclusions	No data was excluded
Replication	Unless otherwise indicated, all data was acquired in three independent experiments.
Randomization	For the majority of data in the paper, no randomisation was performed. For in-vivo experiments, mice were randomly assigned to cages on arrival for injection with the given cancer cell types. We did not control for covariates as only a single variable was changed (the injected cell line); all animals had the same age, gender, diet and housing conditions.
Blinding	The investigators were not blinded during experiments and outcome assessment, apart from the scoring of IHC data in Figure 6, Figure 7 and Extended Data Figure 10. As acquisition and analysis was performed by the same experimenter, blinding was not deemed necessary.

Reporting for specific materials, systems and methods

We require information from authors about some types of materials, experimental systems and methods used in many studies. Here, indicate whether each material, system or method listed is relevant to your study. If you are not sure if a list item applies to your research, read the appropriate section before selecting a response.

Materials & experimental systems

n/a	Involved in the study	
☐	☒	Antibodies
☐	☒	Eukaryotic cell lines
☒	☐	Palaeontology and archaeology
☐	☒	Animals and other organisms
☐	☒	Human research participants
☒	☐	Clinical data
☒	☐	Dual use research of concern

Methods

n/a	Involved in the study	
☒	☐	ChIP-seq
☒	☐	Flow cytometry
☒	☐	MRI-based neuroimaging

Antibodies

Antibodies used	For western blotting antibodies raised against LAP1 (1:1000; #21459-1-AP) , Lamin A/C (1:1000, #10298-1-AP), Lamin B1 (1:1000, # 12987-1-AP) and Lamin B2 (1:1000, #10895-1-AP) were from Proteintech; pThr18/Ser19-MLC2 (1:750, #3674), MLC2 (1:750, #3672) were from Cell Signalling Technology; GAPDH (1:10,000, #MAB374, clone 6C5) was from Merck. Secondary antibodies were: IRDye 680RD goat anti-rabbit IgG (1:10,000, #925-68071) and IRDye 800RD goat anti-mouse IgG (1:10,000, #925-32210) from LI-COR.<br><br>For immunofluorescence and/or immunohistochemistry, antibodies against Lamin A/C (1:200, #10298-1-AP) from Proteintech or (1:200; #mab3538, clone 131C3) from Millipore; Lamin B1 (1:200, #12987-1-AP), Lamin B2 (1:200, #10895-1-AP), LAP1 (1:200; #21459-1-AP) and Emerin (1:1000, #10351-1-AP) from Proteintech; pSer19-MLC2 (1:200, #3671) from Cell Signalling); HA.11 (1:500, #901503, clone 16B12) from BioLegend; Gamma-H2AX (1:600, #05-636, clone JBW301) from Merck; 53BP1 (1:600, #NB100305) from Novus Biologicals; Mab414 (1:200, #ab24609, clone mAb414), CITED1 (1:200, #ab87978, clone 5H6) and SOX10 (1:500, #ab155279 clone EPR4007) from Abcam; GFP (1:5000, #A-11122) from ThermoFisher. Secondary antibodies were Alexa Fluor 488 (1:1000. A-21202) and Alexa Fluor 555 (1:1000, A-31572) were from Life Technologies.
Validation	Validation relied upon statements from the manufacturer's websites. Anti-LAP1 (#21459-1-AP), anti-Lamin A/C (#10298-1-AP), anti-Lamin B1 (# 12987-1-AP) and anti-Lamin B2 (#10895-1-AP) were additionally validated in this study (Figure ED6C, Figure ED8E) using western blotting and RNA-interference. Anti-MLC2 (#3672) and anti-pMLC (#3674, #3671) have been validated in numerous publications from the V.S.-M. lab (PMID:26464464; PMID:21840487; PMID:18984162; PMID:31935375; PMID:30712866) and are additionally validated on the Cell Signalling Technology website.

Eukaryotic cell lines

Policy information about cell lines

Cell line source(s)	The human melanoma cells WM1361 and WM1366 cell lines were provided by Prof Richard Marais (Cancer Research UK Manchester Institute), who obtained them directly from the original deriver, Prof Meenhard Herlyn; the human melanoma cells WM793B, WM983A, WM983B and WM88 were purchased from the Wistar Collection at Coriell Cell Repository; the human melanoma cells A375P and A375M2 were from Dr. Richard Hynes (HHMI, MIT); the human primary melanocytes M206 and M443 were a kind gift from Dr. Benilde Jiménez (Universidad Autónoma de Madrid and Instituto de Investigaciones Biomédicas CSIC-UAM, Spain) and were isolated from foreskins obtained with informed written consent from healthy donors and under approval of the Institutional Review Board of Hospital Infantil Universitario Niño Jesus (Madrid, Spain); 293T cells were obtained from the Francis Crick Institute's Cell Services Science Technology Platform. CAL-51 cells were kind gifts from Prof Andy Tutt and the Breast Cancer Now Biobank (King's College London and The Institute of Cancer Research, London).
Authentication	Cells were deposited with Crick Cell Services Science Technology Platform and authenticated by STR profiling.
Mycoplasma contamination	Mycoplasma-free vials of cells were recovered from Crick Cell Services Science Technology Platform.
Commonly misidentified lines (See ICLAC register)	No commonly misidentified lines appearing on this register were used in this study.

Animals and other organisms

Policy information about studies involving animals; ARRIVE guidelines recommended for reporting animal research

Laboratory animals	Severe combined immunodeficient SCID (NOD.CB17-Prkdcscid/NcrCrl) mice were obtained from Charles River and NXG (NOD-Prkdcscid-Il2rgtm1/Rj) mice were obtained from Janvier-Labs. Mice were female 6-8 weeks old for all experiments. Animals were housed in the QMUL Biological Services holding facility which maintained a 7 hour light/dark cycle, an ambient temperature of 19-22 °C and humidity of 50-60%.
Wild animals	No wild animals were used in this study.
Field-collected samples	This study did not involve samples collected from the field.
Ethics oversight	All animal experiments were approved by the Ethical Review Process Committees at Barts Cancer Institute & King's College London and carried out under licence from the Home Office, UK.

Note that full information on the approval of the study protocol must also be provided in the manuscript.

Human research participants

Policy information about studies involving human research participants

Population characteristics	Detailed information of the patients included in this study can be found in Extended Data Tables 13 and 14.
Recruitment	Patients diagnosed with melanoma were recruited from the Dermatology departments at Hospital Arnau de Vilanova (Lleida, Spain) and Hospital de Bellvitge (Barcelona, Spain) during the period 1992 to 2014. No selection on age or gender were made. All cases were diagnosed in the respective Pathology Departments as melanoma according to the latest AJCC criteria. Samples were collected with specific informed consent.
Ethics oversight	Tumour samples were processed by IRBLleida (PT17/0015/0027) and HUB-ICO-IDIBELL (PT17/0015/0024) Biobanks integrated in the Spanish National Biobank Network and Xarxa de Bancs de Tumors de Catalunya following standard operating procedures with the appropriate approval of the Ethics and Scientific Committee and were collected with specific informed consent, in accordance with the Helsinki declaration. the human primary melanocytes M206 and M443 were a kind gift from Dr. Benilde Jiménez (Universidad Autónoma de Madrid and Instituto de Investigaciones Biomédicas CSIC-UAM, Spain) and were isolated from foreskins obtained with informed written consent from healthy donors and under approval of the Institutional Review Board of Hospital Infantil Universitario Niño Jesus (Madrid, Spain)

Note that full information on the approval of the study protocol must also be provided in the manuscript.
